# Hepato-Duodenal Fistula Complicating a Pyogenic Liver Abscess: An Unusual Presentation

**DOI:** 10.7759/cureus.12236

**Published:** 2020-12-23

**Authors:** Amtoj Singh Lamba, Bhavneet Singh, Monica Gupta, Swati Dahiya, Ruchika Saini

**Affiliations:** 1 Internal Medicine, Government Medical College and Hospital, Chandigarh, IND; 2 Radiology, Government Medical College and Hospital, Chandigarh, IND; 3 General Medicine, Government Medical College and Hospital, Chandigarh, IND

**Keywords:** liver abscess, pyogenic, klebsiella pneumoniae, fistula, duodenum

## Abstract

An elderly male with type 2 diabetes mellitus was admitted to the emergency ward with fever and pain over the right hypochondrium since one month. An abdominal ultrasound revealed an ill-defined hypoechoic lesion with multiple air foci measuring 8 x 6 x 4 cm within the left lobe of the liver implicating segments III b and IV. A contrast-enhanced computed tomography (CECT) scan of the abdomen showed a similar lesion with leakage of oral contrast into the dependent areas of the collection from a rent in the antero-inferior aspect of the first part of the duodenum. Hepato-duodenal fistula or entero-hepatic fistula secondary to pyogenic liver abscess is an atypical and unusual complication requiring a high degree of suspicion for its diagnosis. Though optimal therapy for its management is still not known, early diagnosis with prompt initiation of therapy is imperative to reduce mortality.

## Introduction

Liver abscess is an intra-abdominal infection characterised by localised collection of pus accompanied by the destruction of hepatic parenchyma [1]. This clinical entity has been described in medical literature since the time of Hippocrates. It may be of bacterial, fungal or parasitic origin with Escherichia coli being the most frequently isolated organism till the 1980s and 1990s [2]. In recent decades, Klebsiella pneumoniae has emerged as an important causative agent of liver abscess world-wide with predominance in the Southeast Asia region [2]. Diabetes mellitus is a significant risk factor implicated in K. pneumonia-related liver abscess, with poor glycaemic control resulting in worse prognosis and complications [2]. Liver abscess if diagnosed early with prompt initiation of therapy has reduced the chances of it progressing towards various complications and subsequent mortality. Mortality from liver abscess is low if it is confined to the liver with the right lobe of the liver being the most common site of localization in 75% of the cases [1]. However, when an abscess ruptures and extends into adjacent structures, it leads to the development of complications thereby increasing the mortality rate. Involvement of the thoracic, peritoneal or pericardial cavity following rupture is often noted but extension into the gastrointestinal tract is rare and unusual. With only a limited number of reports on complications in pyogenic liver abscess, we present a recently encountered rare and unusual case of pyogenic liver abscess. The abscess was found to be complicated by the formation of a fistulous tract with duodenum, which was confirmed by leakage of oral contrast from the duodenum into the hepatic collection on contrast-enhanced computed tomography (CECT) of the abdomen.

## Case presentation

A 71-year-old male with diabetes mellitus of seven-year duration was admitted to the emergency ward with the primary complaints of one month of mild-to-moderate fever and dull aching abdominal pain which was localised over the right upper abdomen. The patient gave no history of associated nausea, vomiting, jaundice, diarrhea, dysentery or weight loss. No history of similar episodes in the past. The patient was shifted from oral hypoglycemic agents to insulin therapy one and a half years back because of his uncontrolled blood glucose levels; however, he was not compliant to insulin therapy. Around the same time, he developed an ulcer over the left medial malleolus for which debridement and split skin grafting was done.

At presentation, the patient was conscious and well oriented. He was febrile (101.4°F) and his blood pressure read 80/50 mmHg, heart rate 118/min and oxygen saturation of 97% under room air. The cardiovascular system revealed normal heart sounds and no murmurs were heard. He had severe pallor and tenderness on palpation over the right hypochondrium, with rest of the systemic examination being unremarkable. 

Laboratory investigations revealed a random blood glucose of 286 mg/dl, anemia with haemoglobin of 4.2 gm/dl, raised total leukocyte count of 23.5 X 10^9^/L with 94% neutrophils. Erythrocyte sedimentation rate was 86 mm/first hour. The total serum bilirubin was 1.2 mg/dl with an alkaline phosphatase of 139 IU/L and aspartate aminotransferase and alanine aminotransferase of 58/33 IU/L respectively. Blood urea and serum creatinine levels were 97mg/dl and 1.5mg/dl respectively. Glycosylated haemoglobin was 12.1% and he had traces of albumin in urine. Electrocardiogram and chest X-ray were normal. An abdominal ultrasound revealed an ill-defined hypoechoic lesion measuring 8 x 6 x 4 cm contained within the left lobe of the liver involving segments III and IV b along with multiple air foci. Hydatid serology for Echinococcus granulosus was negative. Blood and urine cultures did not show growth of any organism.

**Figure 1:**
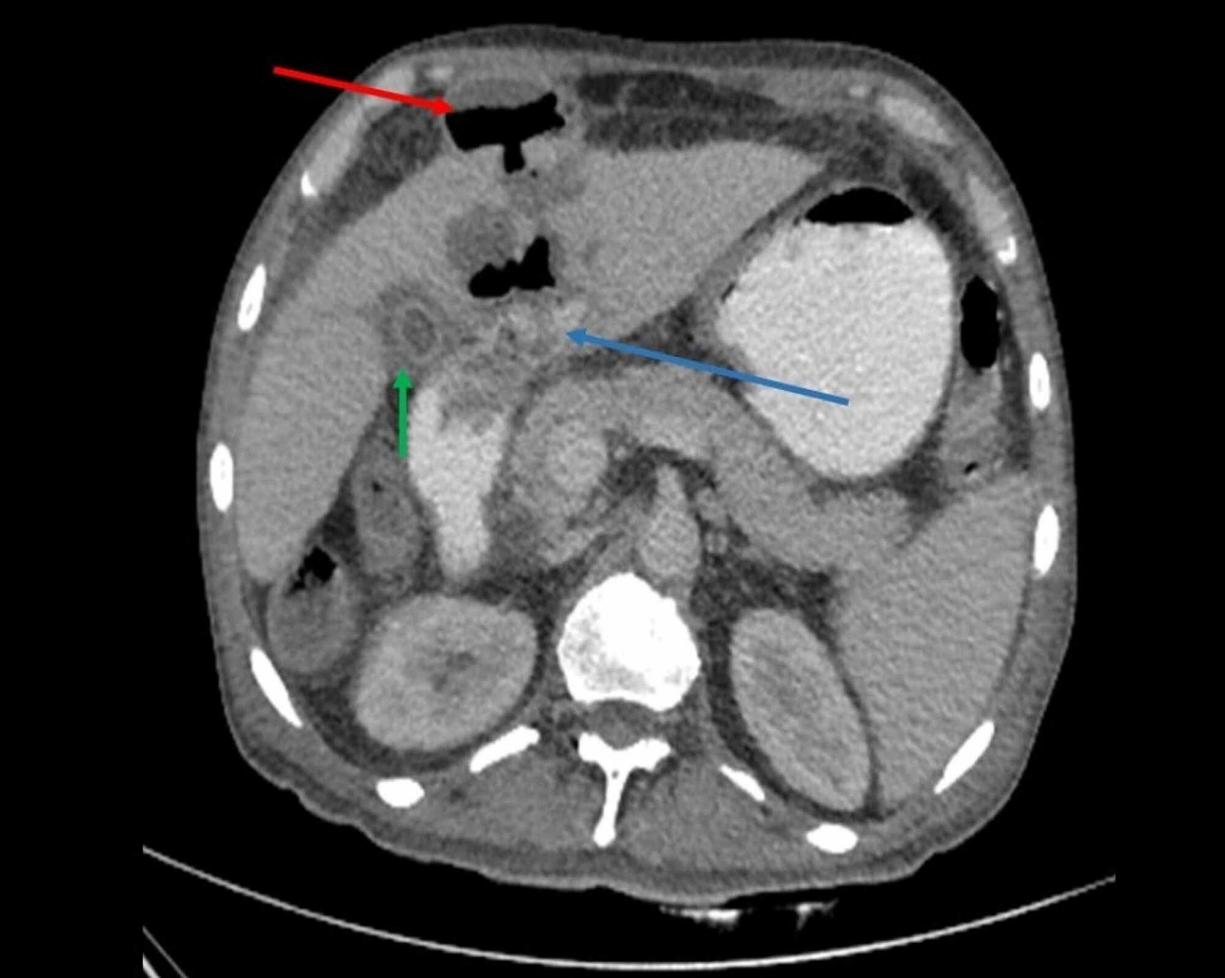
Axial contrast-enhanced CT abdomen showing extravasation of oral contrast into liver abscess (blue arrow). Note the reactive edematous thickening of gall bladder (green arrow) and pre-peritoneal rupture of abscess (red arrow).

**Figure 2:**
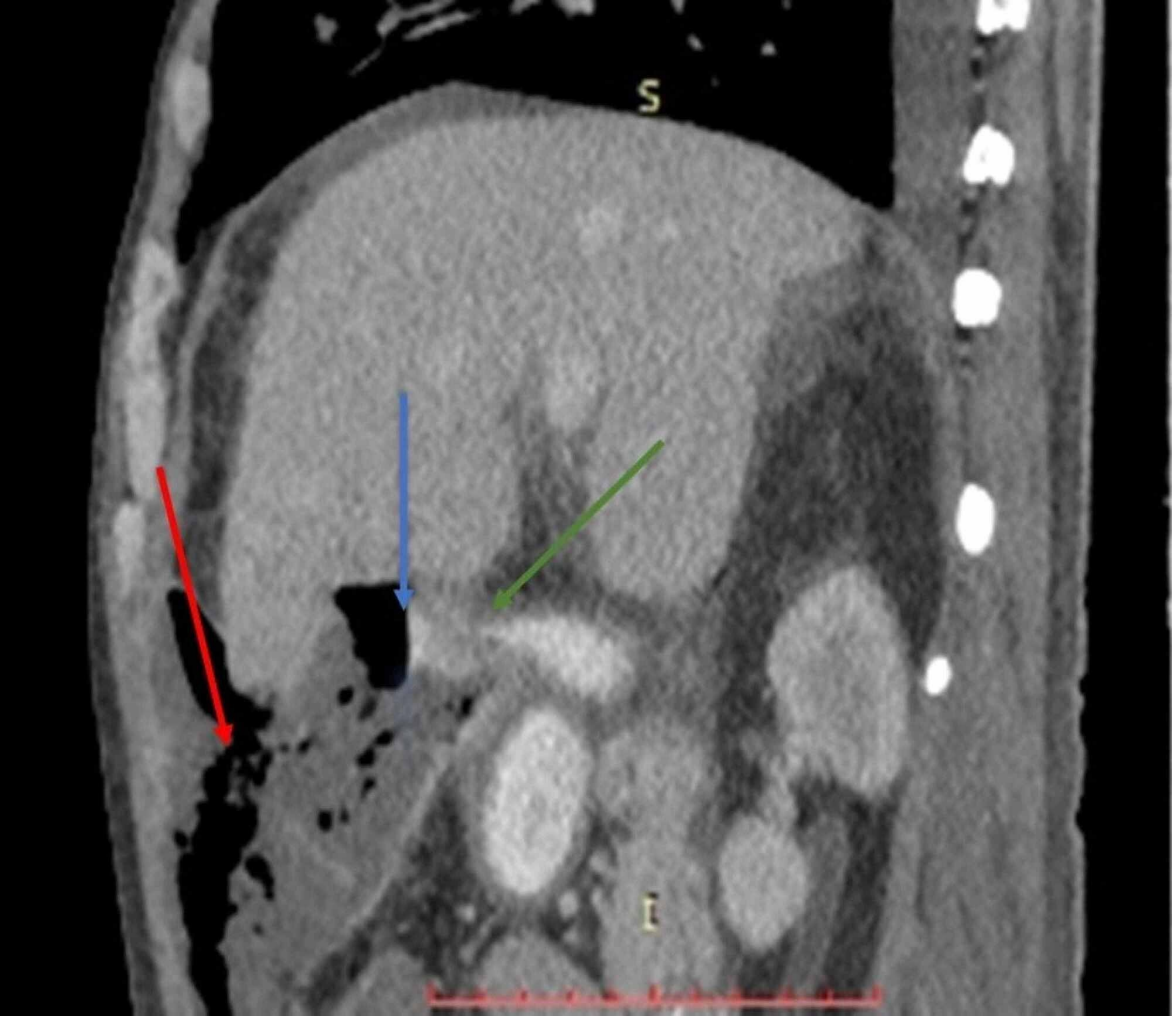
Sagittal reconstruction of contrast-enhanced CT of upper abdomen showing a contrast filled fistulous tract connecting the air containing liver abscess and first part of duodenum (green arrow). Note the contrast fluid level within abscess (blue arrow) and anterior rupture of abscess in pre-peritoneal space (red arrow).

After instituting the initial resuscitative measures with fluids and inotropic support and initial empirical antibiotic administration, a diagnostic aspiration was carried out under ultrasound guidance. Frank pus was aspirated and sent for culture sensitivity. A contrast enhanced CT scan of the abdomen was done which revealed a well-defined irregular peripherally enhancing heterogeneously hypodense collection measuring 4.6 x 5.2 x 6.9 cm in segment IVb of the liver. Multiple air foci were seen within it. Posteriorly, there was evidence of a rent in the anteroinferior aspect of the first part of duodenum (part of duodenum extending from pyloric end to superior duodenal flexure). Positive oral contrast was seen to be leaking into the dependent areas of the collection suggestive of a hepatoduodenal fistula (Figures 1, 2).

The pus culture grew two organisms namely Enterococcus faecalis and Klebsiella pneumonia and the patient was treated with parenteral antibiotics as per the pus culture sensitivity report; linezolid and piperacillin-tazobactam for a period of two weeks. Supportive treatment included intravenous fluids, inotropic support and transfusion of three units of packed red blood cells. As the patient responded to treatment and his symptoms resolved, he was discharged after 14 days of hospital admission on oral antibiotic therapy for another two weeks. On follow-up, one year after discharge, the patient is asymptomatic and continues to lead a healthy life. A follow-up scan of the abdomen did not reveal evidence of hepatic abscess suggesting spontaneous resolution of the abscess and fistula.

## Discussion

Liver abscess, depending upon the causative organism can broadly be classified into amoebic and pyogenic, where pyogenic liver abscess is further classified into bacterial or fungal [3]. Previously, portal pyemia following intra-abdominal infections resulted in the majority of cases of pyogenic liver abscess, however with the rampant use of antibiotics most reports are now said to be cryptogenic. These cases are common in men and in the older population with underlying diabetes or other predisposing states such as malignancy, previous biliary surgery or immunosuppressive states [4]. Entamoeba histolytica is a pathogenic amoeba infecting the gastrointestinal tract and amoebic liver abscess is a frequent extra-intestinal manifestation. It is commonly prevalent in subtropical and tropical areas where overcrowding, poor sanitation and undernutrition are common.

Pyogenic liver abscess has an incidence rate ranging from 0.029% to 1.47% for hospitalised patients [5]. Patients commonly present with epigastric or right upper quadrant (RUQ) pain, low-grade fever, anorexia and malaise with elicitable tenderness over RUQ, hepatomegaly or palpable mass along with jaundice and ascites [6]. Amoebic liver abscess has a reported complication rate of 10.3% [7]. However reports on complications arising in a pyogenic liver abscess are handful, with a fistula formation between a hepatic abscess and gastrointestinal tract being an extremely rare occurrence [6]. 

For patients presenting with chief complaints of fever and right upper quadrant pain of one-month duration other differentials such as cholecystitis, pancreatitis, peri hepatic collection, chronic duodenal ulcer with localised perforation, mesenteric ischemia, etc., should be kept in mind and ruled out using appropriate investigations. In our case the patient gave no history of steroids, analgesics (NSAIDs) or antimalarial ingestion, no post-prandial abdominal pain and on palpation had no guarding, rigidity or rebound tenderness. Plain X-ray abdomen was not suggestive of air under diaphragm and the ultrasound abdomen neither revealed any free fluid in the peritoneal cavity nor did it detect the presence of gall stones or bulky pancreatic head.

An abdominal ultrasound is a rapid and a non-invasive tool for the diagnosis of liver abscess. Detection of air in the abscess cavity should caution the clinician to rule out the possibility of either a hepatobronchial or a hepatoenteric fistula or a secondary bacterial infection complicating a liver abscess. To confirm the presence of a hepatoenteric fistula, a water-soluble barium swallow and/ or CT with oral contrast is a pertinent radiological investigation [8]. The common sites of rupture for a liver abscess into the gastrointestinal tract are the stomach and the duodenum and rarely it may be associated with rectal passage of pus [1].

Enterohepatic fistula is an acquired variety of gastrointestinal internal fistula. In our patient, it resulted from the rupture of liver abscess with erosion of adjacent duodenal wall. Suspicion was raised because of the presence of air in the abscess cavity on ultrasonography following which the fistulous communication could be visualised on cross-sectional imaging, i.e., CECT scan of the abdomen [9].

In a retrospective analysis of 81 patients with pyogenic liver abscesses, 21 patients developed complications with only one patient developing rupture of abscess into the gastrointestinal tract [1]. Ever since it was first described in 1983, only 13 reports have been published till date on rupture of a liver abscess with subsequent fistulisation into the gastrointestinal tract thereby signifying that it is an extremely rare complication [6].

With no well-defined guidelines outlined for the management of this complication, surgical management is considered definitive however conservative and supportive measures may assist in spontaneous closure of fistulas. A study that highlighted a hepato-gastric fistula complicating a pyogenic liver abscess due to a delay in its percutaneous drainage also implicated the role of early abscess drainage so as to preclude such complications [10].

## Conclusions

The present case draws attention towards an entero-hepatic fistula secondary to pyogenic liver abscess. It is an atypical and unusual complication requiring a high degree of suspicion for its diagnosis. Though optimal therapy for its management is still not known, high-resolution imaging modalities such as ultrasonography, CT scan and image-guided percutaneous drainage have not only rendered a critical role in its early diagnosis but have also greatly diminished the mortality rate thereby improving patient care.
